# Recurrent Violent Behavior: Revised Classification and Implications for Global Psychiatry

**DOI:** 10.3389/fpsyt.2017.00151

**Published:** 2017-08-28

**Authors:** Karen Herrera-Ferrá, James Giordano

**Affiliations:** ^1^Asociación Mexicana de Neuroética, Mexico City, Mexico; ^2^Departments of Neurology and Biochemistry; Neuroethics Studies Program-Pellegrino Center for Clinical Bioethics, Georgetown University Medical Center, Washington, DC, United States

**Keywords:** violence, ethics, psychiatry, human rights, global mental health

## Introduction

Interpersonal violent acts are a worldwide problem. Deterrence of violence has been, and remains a complicated and often unsuccessful enterprise, given that violence is multi-factorial, and occurs in a variety of contexts (e.g., domestic violence; serial killing; mass slaying; terrorism; collective violence), and on a global scale within diverse socio-cultural populations (1). Given this broad impact, there have been, and continue to be public and political calls—if not demands—to employ research findings to both better identify and mitigate or prevent causes and occurrences of violent behavior (2–4). Current research and interventions in violent behavior are mainly focused upon four domains: (a) attention to the trauma and needs of victims (5), (b) recidivism of violent crime (6), (c) violence as an expressed trait of certain neuropsychiatric disorders (7), and (d) seeking predictive variables (8).

Previously, we have suggested that *recurrent violent behavior* (RVB)^1^ should be considered as a psychiatric classifier that can be employed to initiate further medical and public safety interventions within an interdisciplinary approach, as we acknowledge that RVB is often multi-etiologic, and involves biological as well as psychosocial factors (9). In this light, we have explored the viability of using RVB in *DSM* and/or *ICD* frameworks, which could be applicable in developed as well as developing and non-developed countries’ as a component of the proposed global mental health (GMH) plan (10). We believe that this could yield novel opportunities for studying chronic violence as an international mental health issue. However, we also note the potentially controversial nature of such classification, and acknowledge the need to prudently address validity, viability, benefits, risks, and harms that could be incurred by using neuroscience and neurotechnology to assess and intervene against recurrent violence (11).

## Frequency of Violence: Viability as a Pathological Trait?

Violence is part of the human behavioral repertoire; but while the propensity for violence may be considered to be “normal,” the actual execution of violence—and particularly recurrent violence—is regarded as a maladaptive reactive behavior (12, 13) that manifests socio-legal disruption. Apropos such regard, we support that when evaluating the relative normality of a trait or characteristic, intensity, and/or frequency must be considered (12, 13). Intensity of the violent act is mainly represented by the level of harm, and such acts generally incur response by legal authorities. However, “non-visible” harm, often viewed as “less intense” conduct (e.g., certain forms of psychological/domestic violence), frequently do not prompt legal or even medical intervention because “…*medical professionals do not perceive that is part of their role to investigate suspected domestic/psychological violence*…” (14–16).

Thus, we posit that *frequency* (i.e., recurrence) should be viewed as a hallmark feature of a harmful pattern of actions that is interpersonally disruptive and deviant from prescribed social norms and broadly expected behavioral control (9). Given this definition, we assert that the paucity or absence of medical/psychiatric care of persons with RVB jeopardizes mental health, public safety, and undermines the ethical probity of medicine as an individual and public good. So, if typified as a psychiatric classifier, RVB (as a behaviorally overt trait) could be observed, depicted, assessed, diagnosed, and medically engaged, regardless of intensity and/or etiology.

## GMH and Human Rights: Re-Classifying RVB as Important to a Combined Agenda

Our proposed aim of instating RVB as a psychiatric classifier is to leverage medical and psychosocial interventions in order to improve both mental health care and public safety needs, as consistent with stated directives of Article 25th of the United Nations Universal Declaration of Human Rights (17), and World Health Organization (WHO) Mental Health Action Plan 2013–2020 (18). At present, interpersonal RVB is regarded as an expressed trait of certain neuropsychiatric disorders (e.g., conduct disorder, psychosis, personality disorders, brain tumor, etc.) (12, 13), but we feel that this diagnostic classification is limited, and constrains (1) access to psychiatric care, (2) mental health of the recurrently violent individual, and (3) efforts in and provision of public safety.

However, we acknowledge that simply instituting RVB as a psychiatric classifier does not necessarily guarantee proper—if any—medical assessment or intervention, and this may be especially true in developing and undeveloped countries. At present, 90% of global neuropsychiatric research is performed in 10% of the mental health population of westernized, educated, industrialized, rich, and democratic (WEIRD) countries (19), while 85% of the global population lives in low and middle-income (LAMI) countries (20). This discrepancy in research populations misrepresents global psychiatric epidemiology, and creates a significant gap in knowledge of mental health, and in the viability, delivery, and effectiveness of assessment and care of mental disorders. The GMH initiative specifically seeks to “… *improve services for people living with mental health problems and psychosocial disabilities worldwide, especially in low- and middle- income countries*… *based on scientific evidence and human rights*… *not only for treatment, but for prevention and promotion of mental well being* (10).”

We believe that for RVB to be included in the GMH project as a psychiatric classifier, international research efforts will be required to adapt current, and newly developing assessment and treatment tools to particular socio-cultural ecologies. To do so will necessitate review and revision of mental health resources that are available within—and for—different (developed, developing, and non-developed) countries. This will require collaboration in global research and clinical efforts. As well, the identified global burden of RVB, which undergirds its prioritization as both a mental health and public safety issue, further necessitates detailed consideration of which (and what extent of) resources should be allocated and dedicated to evaluation and intervention. This is particularly true if emerging neurotechnological tools, and systems of medical surveillance are to be employed.

Here, a concern is that WEIRD and more progressively developing countries may exploit economic advantages and relationships to foster greater, and more sustainable efforts in such pursuits. In this scenario, it is possible—if not probable—that the public health and safety burdens incurred by the occurrence, prevalence, and effects of RVB would become increasingly manifest in LAMI countries. This would induce evident mental health, social, economic, and political instability which would only exacerbate extant discrepancies in GMH. Therefore, we propose that a subsequent, logical step would be to incorporate a definition, and recommendations for resources and services necessary for research, assessment, and intervention of RVB as a psychiatric classifier within the scope and activities of the GMH project.

## Ethical Issues Relevant to GMH

We have already sought to identify basic ethico-legal and social issues (ELSI) generated by establishing RVB as a psychiatric classifier (9). However, if positioned within the larger agenda of the GMH project, additional ELSI can, and likely will arise. For example, cultural neurocognitive diversity may render certain forms of violent behavior(s) to be representative of longstanding and generally tolerated cultural actions. While respecting socio-cultural variability, we posit that like many other mental health conditions, diagnostic criteria for RVB could be validated and legitimized across cultures even in light of socio-cultural variance. Thus, RVB may represent and RVB may represent a widely accepted abnorm when: (1) considered to be maladaptive or threatening the wellbeing of others within cultural standards of ethics and/or law and (2) viewed relative to WHO identification of violence as an urgent public health concern worthy of United Nations International Children’s Emergency Fund (UNICEF) and United Nations Office on Drugs and Crime (UNODC) efforts in individual and public safety (1, 12, 13, 21, 22). In this way, if considered a GMH issue, RVB could be formally leveraged so as to facilitate psychiatric assessment and intervention to mitigate, if not prevent further, and increasing occurrence.

However, this may also pose risk of additionally medicalizing if not pathologizing social behavior(s). There has been—and continues to be—debate about the utilization of psychiatry as a tool that can be employed to advance public or political interests in establishing criteria for cognitive, emotional, and behavioral normality and abnormality (23–25). Concepts such as psycho-politics or political psychiatry have emerged in an attempt to describe the use of psychiatric knowledge, assessment and interventions for public policy, prosecution, and “rehabilitation” of individuals who hold certain beliefs and/or effect particular actions (26).

Clearly, this has potential for significant abuse and harm, and the use of diagnostic labels as substantiation for individuals’ behaviors and/or to justify legal action has been discussed elsewhere (9, 27). Hence, caution must be taken when formulating specific criteria for the definition and use of RVB in particular (ethno) psychiatric contexts within a GMH agenda. Calls for an ethically sound GMH are noteworthy and of value (19), but there is much work to be done if this is to be enacted in practice, and such efforts will require both dedicated resources and the fiscal support to sustain them.

## Conclusion

Establishing RVB as a psychiatric classifier could be a viable and valuable approach toward enabling more accurate assessment and intervention, which could afford both medical and social benefit(s). Moreover, instantiating RVB as a classifier within the ICD framework axiomatically prompts consideration of those ways that this new classification could—and should—be leveraged within a GMH agenda. Inclusion within the GMH project could improve and enable research, availability, access and quality of resources, and services for assessing and treating RVB.

Yet, attempting to medically approach a potentially harmful human conduct poses questions of meanings of normality and socio-cultural variance, and can foster misuse of diagnoses for legal and/or political ends. As well, discrepant economics of WEIRD and LAMI countries may incur issues of unequal and/or inequitable availability and affordability of mental health services, and non-sustainability of care and social benefit. Therefore, we believe that global efforts to engage this approach—and others—to proactively foster mental health and social safety should be an opportunity for collaborative dialogues between developed, developing, and non-developed countries, and should reflect ongoing consideration and articulation of ELSI fostered in and by such efforts. Thus, a more comprehensive discourse—involving internationally representative participants—is needed to most effectively foster and advance such initiatives.

## Author Contributions

KH-F provided substantial contributions to the conception, design, drafting the work, and revising it critically for important and significant intellectual content as well as final approval of the version to be published. She has agreed to be accountable for all aspects of the work in ensuring that questions related to the accuracy or integrity of any part of the work are appropriately investigated and resolved. JG provided substantial contributions to the conception, design, drafting the work, and revising it critically for important and significant intellectual content as well as final approval of the version to be published. He has agreed to be accountable for all aspects of the work in ensuring that questions related to the accuracy or integrity of any part of the work are appropriately investigated and resolved.

## Conflict of Interest Statement

The authors declare that the research was conducted in the absence of any commercial or financial relationships that could be construed as a potential conflict of interest.

## Footnotes

^1^Maladaptive, repetitive, and persistent pattern of intentional use of physical force or power, threatened or actual, against oneself, another person, or against a group or community, which either results in or has a high likelihood of resulting in physical injury, psychological or social harm, maldevelopment, or death. The pattern represents a clinically significant disturbance in an individual’s emotional regulation that reflects dysfunction in the biological, psychological, social, and/or developmental processes involved in cognitive and behavioral functioning (9).
